# Correction: Critical role for Sec22b-dependent antigen cross-presentation in antitumor immunity

**DOI:** 10.1084/jem.2017022902092018c

**Published:** 2018-03-05

**Authors:** Andrés Alloatti, Derek C. Rookhuizen, Leonel Joannas, Jean-Marie Carpier, Salvador Iborra, Joao G. Magalhaes, Nader Yatim, Patrycja Kozik, David Sancho, Matthew L. Albert, Sebastian Amigorena

Vol. 214, No. 8, August 2017. https://doi.org/10.1084/jem.20170229

The authors regret that in the original version of their paper, they mistakenly used the phrase OVA-expressing cells instead of OVA-secreting cells in parts of the text and cited reference Boissonnas et al. (2007. http://dx.doi.org/10.1084/jem.20061890) instead of Zeelenberg et al. (2008. http://dx.doi.org/10.1158/0008-5472.CAN-07-3163) and Sedlik et al. (2014. http://dx.doi.org/10.3402/jev.v3.24646). The Results and discussion paragraph containing the corrected references and full bibliographic information appear below:

“To address the possible role of Sec22b-dependent antigen cross-presentation in tumor immunotherapy by checkpoint blocking antibodies, we used a less-immunogenic tumor, OVA-secreting MCA-101 (Zeelenberg et al., 2008; Sedlik et al., 2014). This tumor is well controlled by antibodies against CTLA-4 or PD-1 (Gubin et al., 2014). Sec22b^−/−^ and Sec22b^+/+^ mice were injected with tumor cells and received or did not receive anti–PD-1 treatment, as described previously (Gubin et al., 2014). As expected, treatment with anti–PD-1 induced tumor rejection in Sec22b^+/+^ littermates (Fig. 4 E). In contrast, Sec22b^−/−^ mice failed to respond to anti–PD-1 treatment, both in terms of tumor growth and survival (Fig. 4, F and G). Consistently, treatment with anti–PD-1 induced an anti-OVA immune response in littermates, but not in Sec22b^−/−^ mice, when analyzed by T cell restimulation and subsequent secretion of IFN-γ (Fig. 4 H and Fig. S3 B).”

Sedlik, C., J. Vigneron, L. Torrieri-Dramard, F. Pitoiset, J. Denizeau, C. Chesneau, P. de la Rochere, O. Lantz, C. Thery, and B. Bellier. 2014. Different immunogenicity but similar antitumor efficacy of two DNA vaccines coding for an antigen secreted in different membrane vesicle-associated forms. *J. Extracell. Vesicles*. 3:24646. http://dx.doi.org/10.3402/jev.v3.24646

Zeelenberg, I.S., M. Ostrowski, S. Krumeich, A. Bobrie, C. Jancic, A. Boissonnas, A. Delcayre, J.-B. Le Pecq, B. Combadière, S. Amigorena, and C. Théry. 2008. Targeting tumor antigens to secreted membrane vesicles in vivo induces efficient antitumor immune responses. *Cancer Res.* 68:1228–1235. http://dx.doi.org/10.1158/0008-5472.CAN-07-3163

The online HTML and PDF versions of this paper have been corrected. The errors remain only in the print version.

